# Vestibulo-ocular reflex dynamics with head-impulses discriminates Usher patients type 1 and 2

**DOI:** 10.1038/s41598-024-54270-y

**Published:** 2024-02-14

**Authors:** Ana Margarida Amorim, Ana Beatriz Ramada, Ana Cristina Lopes, Eduardo Duarte Silva, João Lemos, João Carlos Ribeiro

**Affiliations:** 1 Department of Otorhinolaryngology, Coimbra University Hospital Centre, Praceta Mota Pinto, 3000-135 Coimbra, Portugal; 2 Faculty of Medicine, University of Coimbra, Coimbra, Portugal; 3 Department of Ophthalmology, Central Lisbon Hospital Centre, Lisbon, Portugal; 4 Department of Neurology, Coimbra University Hospital Centre, Faculty of Medicine, University of Coimbra, Coimbra, Portugal

**Keywords:** Genetics, Medical research

## Abstract

Usher Syndrome classification takes into account the absence of vestibular function but its correlation with genotype is not well characterized. We intend to investigate whether video Head Impulse Test (vHIT) is useful in screening and to differentiate Usher Syndrome types. 29 Usher patients (USH) with a genetically confirmed diagnosis and 30 healthy controls were studied with vHIT and dizziness handicap inventory questionnaire (DHI). Statistical significant differences between USH1, USH2 and controls were found in the vestibulo-ocular-reflex (VOR) gain of all SCCs, with USH1 patients consistently presenting smaller gains. VOR gain of the right lateral SCC could discriminate controls from USH1, and USH2 from USH1 with an overall diagnostic accuracy of 90%. USH1 DHI correlated with VOR (ρ = − 0,971, *p* = 0.001). Occurrence rate of covert and overt lateral semicircular canals refixation saccades (RS) was significantly different between groups, being higher in USH1 patients (*p* < 0.001). USH1 peak velocity of covert and overt saccades was higher for lateral semicircular canals (*p* < 0.05 and *p* = 0.001) compared with USH2 and controls. Covert saccades occurrence rate for horizontal SCCs could discriminate USH1 from USH2 patients and controls with a diagnostic accuracy of 85%. vHIT is a fast and non-invasive instrument which allowed us to screen and distinguish Usher patients from controls with a high precision. Importantly, its use allowed further discrimination between USH1 from USH2 groups. Moreover, VOR gain seems to correlate with vertigo-related quality of life in more severe phenotypes.

## Introduction

Usher syndrome (USH) encompasses a group of rare autosomal recessive ciliopathies characterized by the association of sensorineural hearing loss, rod-cone dystrophy, and in some cases, vestibular dysfunction with a worldwide prevalence of 4–17:100,000^1^. It is commonly separated into three clinical phenotypes: type 1 (USH1), diagnosed in the first decade of life, with profound sensorineural hearing loss (SNHL) and congenital vestibular dysfunction; type 2 (USH2), diagnosed in early adulthood, SNHL and normal vestibular function; and type 3 (USH3), diagnosed in the first decade of life, with progressive deafness and/or variable expression of vestibular dysfunction^2^. Nine confirmed genes correlate with USH1, USH2, USH3 and at least another 8 genes are still to be confirmed, including one responsible for USH 4^3,4^.

While USH classification takes into account the vestibular function, it is infrequently studied. When assessments are conducted, they often involve prolonged and uncomfortable tests^5–9^. The video HIT (vHIT) is a non- invasive quick bedside test (~ 10 min), which allows the evaluation of each semicircular canal (SCC) function. Namely, it quantifies the vestibulo-ocular reflex (VOR) gain and further enables the characterization of the catch-up saccades triggered by head impulses. These can be generated during (ie, covert saccades) and/or after (ie, overt saccades) the head impulse^10^. vHIT use to study vestibular function in Usher patients is not well described^5,11,12^.

Here we investigated whether VOR dynamics when explored with the vHIT are useful to differentiate the most common types of USH.

## Material and methods

This observational prospective cohort study was conducted at a tertiary hospital from 2020 to 2022. We consecutively recruited 29 genetically confirmed Usher Syndrome (USH) patients from the Otorhinolaryngology outpatient clinic. The USH cohort comprised USH type 1 (n = 10, 5 female, age = 27.1 ± 15.5 [9 to 57 years]), type 2 (n = 18, 7 female, age = 52.9 ± 14.0 [37 to 82 years]), and type 4 (n = 1, male, 61 years old).

Additionally, 30 healthy individuals (24 female, age = 45.3 ± 17.4 [9 to 82 years]) were recruited from hospital staff and patient caregivers to serve as a control group. This group was selected to encompass the age range between USH1 and USH2, ensuring age alignment with all patients. Inclusion criteria for the control group required an absence of prior history related to cochlear, vestibular, visual, and/or neurological diseases.

All participants underwent a standardized clinical interview and an audio-vestibular assessment. Demographic information, including age, gender, race/ethnicity, handedness, and educational level, was collected. Medical history focused on the frequency, duration, and severity of dizziness, oscillopsia, vertigo, and falls over the preceding 12 months, along with medication usage. Best-corrected visual acuity (BCVA) and genetic data were obtained from patient medical records.

Functional impact related to dizziness and vertigo was assessed using the Dizziness Handicap Inventory^13^. A neuro-otological exam, including otoscopy, ocular motor examination, assessment for spontaneous nystagmus, and post head-shaking nystagmus, was performed. Additional tests included clinical head thrust testing, Romberg test for quantifying postural sway and Unterberger test for registering postural deviation. A standard clinical pure-tone and speech audiometry (GSI16®) in a soundproof room were done for audiological assessment. Complete vestibular evaluation comprised the video head impulse test (GN Otometrics, Denmark), vestibular-evoked myogenic potential (VEMP) test (Cervical and Ocular VEMPs) (v2010, Neurosoft®, Ivanovo, Russia), bithermal caloric test (Videonystagmography, Ulmer Synapsis®, Marseille, France), and posturography test (Multitest Equilibre, Framiral®, France).

In alignment with the study research objective, the current investigation, we focus on characterization of the vestibulo-ocular reflex performed with the video system (vHIT GN Otometrics®, Denmark). For this test, the patient wore a pair of weightless, tightly fitting goggles on which a small video camera and a half-silvered mirror, which reflects the image of the participant’s right eye into the camera, were mounted. The eye was illuminated by a low-level infrared light-emitting diode. A small sensor on the goggles measured the head movement. Calibration was performed by the user, keeping his or her head still and positioning the left and right laser dots equidistant on each side of the target dot. Following, the user was asked to look at the laser dot alternately on each side of the target, which was placed 1 m ahead. Thenceforth, the examiner moved the participant head side to side through a small angle, while watching the target, thereby allowing a calibration check to ensure that head and eye velocities were overlaid. Once calibrated, participants were taught that they had to stare at the target while the examiner imposed random and unpredictable head impulses, at least 20 for each plane of the canal (pitch, roll, and yaw planes), at an angle of 15–20°, duration 150–200 ms with peak velocity > 150°/s. For Left Anterior Right Posterior (LARP) and Right Anterior Left Posterior (RALP) positions, during the vertical canal testing, the head was rotated 35-45º toward the right or left, whereas gaze was directed, and the head impulse was applied in the plane of the canals. The set-up parameters were the same for each test. At the end of the test, all head velocity stimuli and eye velocity responses were displayed, together with a graph of the calculated VOR gain (ratio of eye velocity to head velocity) for every head rotation.

The parameters included for the analysis were the VOR mean gain and the occurrence rate, latency and peak velocity of covert (saccades that occur after 70 ms and before the head velocity crosses the zero point out of the total head impulse traces) and overt (saccades that occur after the head velocity crosses the zero point out of the total head impulse traces) refixation saccades (RS). The mean gain was obtained from the gain value after each one of the impulses performed and gain asymmetry was calculated between left and right for each canal. All these values were automatically provided by the software and checked by the investigator.

### Statistical analysis

Data distribution normality was examined through the Shapiro–Wilk test, and homogeneity was assessed using the Levene test. A significance level of 0.05 or a symmetry value below 1.96 in both tests guided the determination of parametric test assumptions. Alternatively, non-parametric tests were employed.

A Mann–Whitney U test was employed to assess whether a difference existed for DHI and best corrected visual acuity between USH1 and USH2.

Paired-samples t-tests compared vestibulo-ocular reflex (VOR) gains between semicircular canals (SCCs) within the Usher syndrome (USH) and control groups. Differences in VOR gains between USH patients and controls were evaluated using independent samples t-tests. A one-way analysis of covariance (ANCOVA), with age and vision as covariates, was utilized for comparisons of VOR gains across control, USH type 1, and USH type 2 groups after confirming model assumptions were adequately met.

The non-parametrical ANCOVA equivalent, a Quade’s rank analysis of covariance, was utilized to compare the total refixation saccades occurrence rate, covert saccades, and overt saccades occurrence rate, latencies and peak velocity, across all six SCCs, with age and vision incorporated as covariates.

Correlation between self-reported dizziness handicap and VOR gain was assessed via Spearman’s rank coefficient.

The discriminative ability of VOR gain in the right lateral SCC impulses and the occurrence of covert RS in distinguishing USH patients was assessed using the area under the receiver operating characteristic curve (AUC-ROC). Sensitivity and specificity were determined by identifying the ROC curve's cut-off point with the highest accuracy.

Significance was set at a p-value less than 0.05 across analyses. Statistical analyses were performed using SPSS V.29 software.

### Ethics approval and consent to participate

This study was performed in line with the principles of the Declaration of Helsinki. Informed consent was obtained from all individual participants or from their legal guardians included in the study. Approval was granted by the Ethics Committee of Coimbra University Hospital Centre (OBS.SF.75–2021).

### Consent for publication

Consent for publication was obtained from all individual participants or from their legal guardians included in the study.

## Results

Table 1 shows the subjects’ main clinical characteristics.

**Table 1:** Clinical characteristics of patients with Usher and controls.

Genotype	USH1 (n = 10)			USH2 (n = 18)		USH4 (n = 1)	*p* value	Control (n = 30)
	MYO7A (n = 8)	CDH23 (n = 1)	PCDH15 (n = 1)	USH2A (n = 14)	USH2C (n = 4)	ARSG (n = 1)		
Age, mean (95% CI) Years, max–min	27.1 (16–38.2) (9–57)			52 (45–58.9) (37–82)		61 (61)		45.3 (38.8–51.8) (9–82)
Female, n	5			7		0		19
Retinopathy, n	7			18		1		N/A
Hearing loss, n	10			17		1		N/A
Cochlear implant, n (laterality)	2 (uni); 4 (bil)^|^			0		0		N/A
Vertigo/imbalance, n	8			12		1		N/A
Best Corrected Visual Acuity^1^	0.687 ± 0.329			0.456 ± 0.279			0.057^2^	
DHI score, median (95% CI)	18.5 (12–45)			54 (39–70)		51	0.056^2^	N/A

USH1—Usher subtype 1. USH2—Usher subtype 2. USH4—Usher subtype 4. DHI—Dizziness Handicap Inventory. CI—confidence interval. ^|^—unilateral (uni) or bilateral (bil). NA—Not applicable. .^1^—visual acuity of the better eye converted to a decimal scale, mean ± standard error.^2^-Mann–Whitney test between USH1 and USH2.

### VOR gains in Usher patients Type 1 and type 2

The difference in VOR gain among USH patients subtypes and controls is detailed in Table 2. The mean VOR gain in controls was consistently just below or equal to 1, and a significant asymmetry was observed between the right and left sides, except for the anterior canals (right lateral canal: 1.01 ± 0.09 vs. left lateral canal: 0.93 ± 0.09, *p* = 0.000; right anterior canal: 0.96 ± 0.17 vs. left anterior canal 0.95 ± 0.10, *p* = 0.974; right posterior canal 0.89 ± 0.1 vs. left posterior canal 0.85 ± 0.13, *p* = 0.024).

**Table 2:** VOR gain of Usher patients subtypes and control group.

Gain, mean ± SD		Control (n = 30)	USH1 (n = 10)	USH2 (n = 18)	*p* value	*R* adjusted squared^1^	Control vs USH1	Control vs USH2	USH1 vs USH2	USH1 versus USH2		
							*p* value	*p* value	*p* value	*p* value	*R* adjusted squared	*p* value
Lateral SCC	R	1.019 ± 0.085	0.461 ± 0.289	0.910 ± 0.148	0.000^|^	0.648	0.000^a1^	0.157^a1^	0.00^a1^	0.000^||^	0.536	0.000^a2^
	L	0.938 ± 0.090	0.455 ± 0.255	0.846 ± 0.123	0.000^|^	0.643	0.000^a1^	0.231^a1^	0.000^a1^	0.000^||^	0.583	0.000^a2^
Anterior SCC	R	0.955 ± 0.168	0.545 ± 0.241	0.754 ± 0.175	0.000^|^	0.403	0.000^a1^	0.001^a1^	0.064^a1^	0.056^||^	0.154	0.56^a2^
	L	0.954 ± 0.100	0.564 ± 0.232	0.815 ± 0.137	0.000^|^	0.488	0.000^a1^	0.006^a1^	0.001^a1^	0.001^||^	0.001	0.001^a2^
Posterior SCC	R	0.893 ± 0.101	0.442 ± 0.306	0.746 ± 0.196	0.000^|^	0.485	0.000^a1^	0.059^a1^	0.000^a1^	0.000^||^	0.497	0.000^a2^
	L	0.852 ± 0.132	0.333 ± 0.275	0.695 ± 0.222	0.000^|^	0.495	0.000^a1^	0.055^a1^	0.000^a1^	0.000^||^	0.470	0.000^a2^

R—Right. L—Left; SCC—semicircular canal. CI—confidence interval. USH—Usher patients. SD—standard deviation. ^|^—ANCOVA test adjusted to age. ^a1^—Bonferroni test adjusted to age. ^||^ANCOVA test adjusted to age and best visual acuity ^a2^—Bonferroni test adjusted to age and best corrected visual acuity.

In the USH group (n = 29), gains were significantly smaller when compared to controls, and were also significantly asymmetric for each pair of canals (right lateral canal 0.763 ± 0.30 [*p* = 0.000] vs. left lateral canal 0.72 ± 0.26 [*p* = 0.000], *p* = 0.001; right anterior canal 0.68 ± 0.22 [*p* = 0.000] vs. left anterior canal 0.73 ± 0.21 [*p* = 0.000], *p* = 0.024; right posterior canal 0.64 ± 0.27 [*p* = 0.000], left posterior canal 0.56 ± 0.29 [*p* = 0.000], *p* = 0.007).

**Figure 1:**
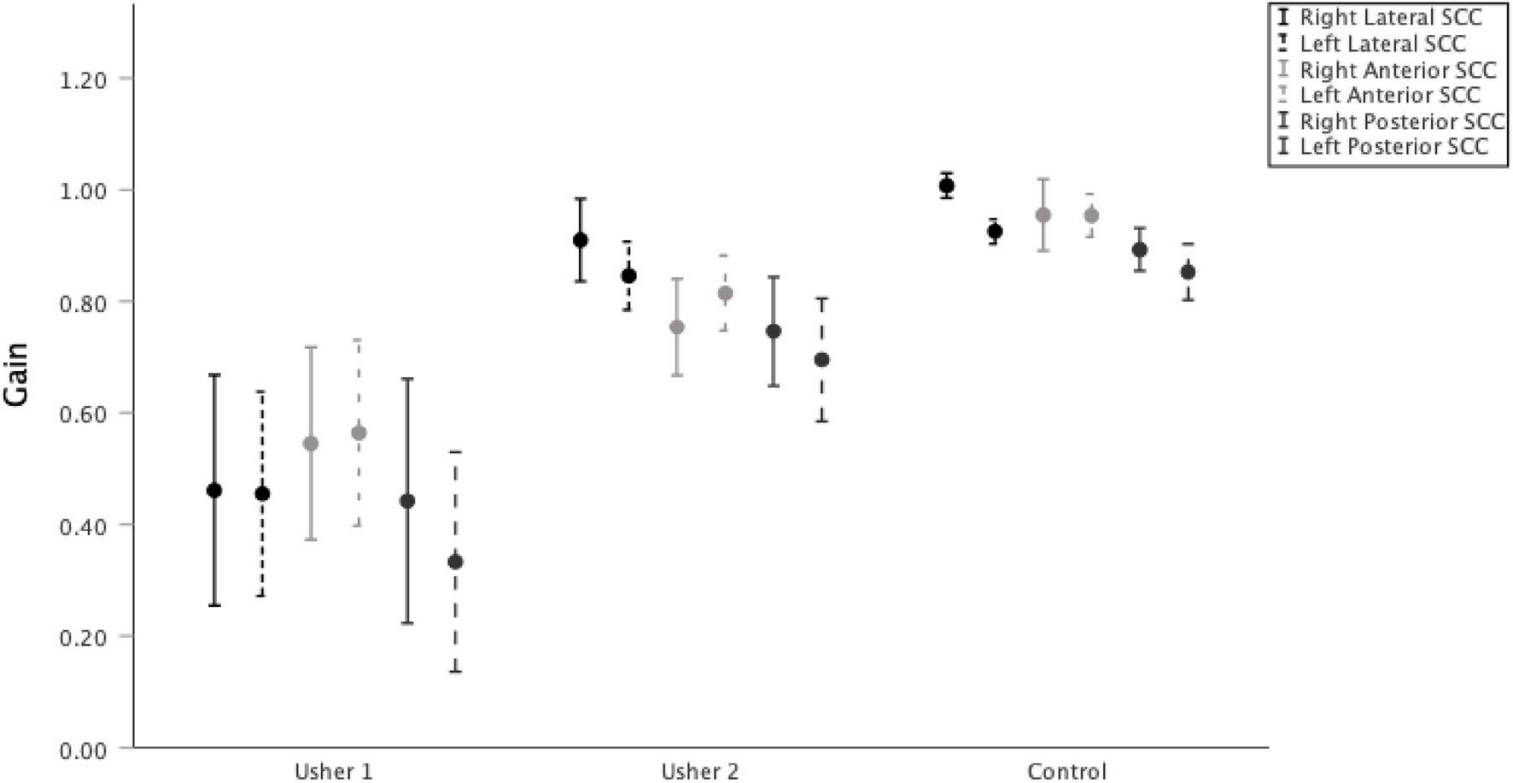
VOR gain of Usher patients subtypes and control group.

The data of USH patients was subsequently analyzed based on its subtypes and compared to controls while controlling for age and vision (only in the USH subgroups). The analysis excluded a single USH4 patient due to the limited sample size. Statistically significant differences between USH1, USH2, and controls were found in the VOR gain for all SCCs (right and left lateral SCC, *p* = 0.000, *p* = 0.000, respectively; right and left anterior SCC, *p* = 0.000, *p* = 0.000, respectively; right and left posterior SCC, *p* = 0.000, *p* = 0.000, respectively). Post hoc tests showed that the VOR gain of the right lateral and left lateral, left anterior and right and left posterior SCCs could differentiate between USH1 and USH2 groups and USH1 and controls. Additionally, the gain of the right anterior SCC could differentiate between USH1 and controls and the gain of the right and left anterior SCC could differentiate USH2 and controls (see Table 2 and Fig. 1 for details).

The VOR gain of the right lateral SCC could discriminate USH1 patients from controls with a diagnostic accuracy of 97,8% (AUC: 0.978). For a VOR gain threshold of 0.775, the sensitivity was 100% and the specificity was 80%. VOR gain of the right lateral SCC could also discriminate USH1 from USH2 patients with an overall diagnostic accuracy of 86.9% (AUC: 0.869). Using a VOR gain threshold of 0.685, sensitivity was 94.4% and specificity was 80% (Table 3).

**Table 3:** ROC Analysis for the gain and the covert saccades in the right horizontal canal to predict differentiation of USH1 from controls and USH1 from USH2.

Variable	Groups	Area	Cut-off value	Specificity (%)	Sensitiviy (%)	Std. Error^a^	Asymptotic Sig^b^	Asymptomatic 95% Confidence Interval	
								Lower bound	Upper bound
VOR gain in R HC	USH1 versus Control	0.978	0.775	80	100	0.019	0.000	0.941	1.000
	USH1 versus USH2	0.869	0.685	80	94.4	0.083	0.001	0.706	1.000
Covert saccades	USH1 versus Control	0.897	14%	96.7	80	0.071	0.000	0.757	1.000
	USH1 versus USH2	0.850	13%	88.9	80	0.082	0.003	0.690	1.000

VOR—Vestibular-Ocular Reflex. R—Right; HC—Horizontal Canal. USH—Usher patients. SD—standard deviation. .^a^- Under the nonparametric assumption. ^b^- Null hypothesis: true area = 0.5

We found a strong negative correlation between total DHI score and left horizontal and right posterior SCC gain for USH1 (Spearman ρ = − 0.812 and ρ = − 0.971, respectively, *p* = 0.05 and *p* = 0.001).

### Characteristics of refixation saccades in Usher patients type 1 and type 2 whilst controlling for age (in three groups) and age and best corrected visual acuity (for USH subgroups)

The total occurrence rate of RS for all the six SCCs except right anterior and right posterior canals was significantly different between groups adjusting for age showing higher rates for USH1 patients versus USH2 patients and controls. When controlling for best corrected visual acuity no significant difference was found between USH 1 and USH2 except for left lateral and left posterior canals (see Table 4 for details).

**Table 4:** Properties and occurrence of refixation saccades in each semicircular canal based on patient subtype.

		Control (n = 30)	USH1 (n = 10)	USH2 (n = 18)	*p* value^1^	*p* value^2^
RS occurrence rate, median (IQR)						
Lateral SCC	R	5 (011)%	59 (31–100)%	23 (5–41)%	0.001^a^	0.23^a2^
	L	0	72 (47–92)%	8 (0–10)%	0.000^a^	0.013^a2^
Anterior SCC	R	0	13 (5–37)%	0	0.217^a^	0.079^a2^
	L	0	14 (0–50)%	0	0.023^a^	0.393^a2^
Posterior SCC	R	5 (0–15)%	20 (12–100)%	8 (5–14)%	0.057^a^	0.341^a2^
	L	0	26 (10–72)%	0	0.000^a^	0.004^a2^
Covert saccades occurrence rate, median (IQR)						
Lateral SCC	R	0 (0–5)%	26 (17–37)%	0 (0–5)%	0.001^a^	0.005^a2^
	L	0 (0–5)%	25 (5–32)%	0 (0–4)%	0.005^a^	0.01^a2^
Anterior SCC	R	0 (0–4)%	0 (0–9)%	0 (0–10)%	0.843^a^	0.929^a2^
	L	0%	0 (0–17)%	0 (0–2)%	0.556^a^	0.927^a2^
Posterior SCC	R	0 (0–8)%	11 (0–27)%	2 (0–5)%	0.192^a^	0.846^a2^
	L	0%	13 (0–22)%	0%	0.02^a^	0.02^a2^
Covert saccades latency, median (IQR)						
Lateral SCC	R	99 (84–108) ms	95 (93–100) ms	94 (88–112) ms	0.740^a^	0.702^a2^
	L	107 (101–112) ms	99 (89–128) ms	98 (89–104) ms	0.186^a^	0.813^a2^
Anterior SCC	R	91 (88–104) ms	104 (87–112) ms	100 (90–106) ms	0.404^a^	0.456^a2^
	L	100 (95–104) ms	93 (72–116) ms	72 ms	0.606^a^	0.419^a2^
Posterior SCC	R	127 (112–156) ms	112 (104–272) ms	127 (92–144) ms	0.755^a^	0.470^a2^
	L	99 (84–144) ms	122 (92–135) ms	90 (80–100) ms	0.694^a^	0.828^a2^
Covert saccades peak velocity, median (IQR)						
Lateral SCC	R	40(33–59)°/s	215 (193–290)°/s	90 (67–208)°/s	0.001^a^	0.088^a2^
	L	49 (45–74)°/s	242 (97–298)°/s	74 (51–132)°/s	0.000^a^	0.126^a2^
Anterior SCC	R	32 (24–40)°/s	44 (37–63)°/s	76 (25–152)°/s	0.168 ^a^	0.212^a2^
	L	41 (14–67)°/s	101 (12–140)°/s	0°/s	0.441^a^	0.607^a2^
Posterior SCC	R	80 (32–93)°/s	153 (99–282)°/s	78 (28–100)°/s	0.071^a^	0.157^a2^
	L	84(57–93)°/s	187 (71–263)°/s	70 (33–107)°/s	0.632^a^	0.954^a2^
Overt saccades occurrence rate, median (IQR)						
Lateral SCC	R	0 (0–12)%	38 (18–84)%	11 (5–31)%	0.001^a^	0.05^a2^
	L	0 (0–5)%	55 (12–78)%	5 (0–21)%	0.000^a^	0.053^a2^
Anterior SCC	R	0%	5 (4–13)%	0%	0.001^a^	0.005^a2^
	L	0%	2 (0–23)%	0%	0.011^a^	0.487^a2^
Posterior SCC	R	0 (0–15)%	10 (9–77)%	5 (5–18)%	0.065^a^	0.305^a2^
	L	0%	13 (0–16)%	0 (0–4)%	0.006^a^	0.059^a2^
Overt saccades latency, median (IQR)						
Lateral SCC	R	240 (192–290) ms	253 (213–317) ms	290 (189–363) ms	0.814^a^	0.548^a2^
	L	232 (152–296) ms	237 (212–316) ms	288 (128–310) ms	0.793^a^	0.342^a2^
Anterior SCC	R	304 (280–508) ms	184 (136–412) ms	299 ms	0.575^a^	0.115^a2^
	L	520 ms	318 (247–384) ms	422 (332–512) ms	0.567^a^	0.696^a2^
Posterior SCC	R	225 (160–324) ms	217 (198–316) ms	332 (266–440) ms	0.026^a^	0.082^a2^
	L	305 (328–479) ms	247 (199–298) ms	301 (192–388) ms	0.745^a^	0.326^a2^
Overt saccades peak velocity, median (IQR)						
Lateral SCC	R	86 (67–101)°/s	221(92–240)°/s	106 (95–136)°/s	0.003^a^	0.157^a2^
	L	76 (70–82)°/s	199 (172–232)°/s	95 (68–110)°/s	0.001^a^	0.014^a2^
Anterior SCC	R	94 (73–102)°/s	74 (47–136)°/s	56°/s	0.164^a^	0.940^a2^
	L	74°/s	97 (94–158)°/s	101 (97–104)°/s	0.390^a^	0.839^a2^
Posterior SCC	R	97 (82–116)°/s	136 (116–201)°/s	86 (67–113)°/s	0.082^a^	0.079^a2^
	L	96 (87–168)	161 (136–196)°/s	116 (63–152)°/s	0.072^a^	0.194^a2^

RS—Refixation Saccades. R—Right. L—Left. SCC—semicircular canal. IQR –interquartile range. USH—Usher patients. ^a1^—Quade test (non-parametric ANCOVA equivalent test) adjusted for age ^.a2^—Quade test (non-parametric ANCOVA equivalent test) adjusted for age and best corrected visual acuity.

The occurrence rate of covert RS while accounting for age for the right (*p* = 0.001) and left lateral (*p* = 0.005) and left posterior (*p* = 0.02) SCCs, and of overt RS for the right (*p* = 0.001) and left lateral (*p* = 0.000), right (*p* = 0.001) and left (*p* = 0.011) anterior and left posterior (*p* = 0.006) SCCs was significantly different between groups, being higher in USH1 patients. When comparing these covert RS and overt RS between USH subtypes adjusting for age and best corrected visual acuity, we encountered the same significant differences for the rate of covert RS for the right (*p* = 0.005), left lateral (*p* = 0.01) and left posterior (*p* = 0.02) impulses. As for the overt RS only the right anterior (*p* = 0.005) impulses showed significant differences.

The peak velocity of covert saccades was higher for USH1 right and left lateral SCC (*p* = 0.001 and *p* = 0.002), while adjusting for age between the three groups, but not when adjusting for age and best corrected visual acuity in USH subtypes. For the peak velocity of overt saccades adjusted for age, USH1 had the higher values for the right and left lateral SCC (*p* = 0.003 and *p* = 0.001) but when adjusting for age and best corrected visual acuity, only the peak velocity of the overt saccades for the left lateral impulses had significant difference between USH subtypes (*p* = 0.014).

We found no differences between groups regarding covert saccades latencies. In contrast, in overt saccades we found higher latencies for right posterior SCC in USH2 patients (*p* = 0.026) for the comparisons adjusting for age, but not when accounting for age and best corrected visual acuity.

The diagnostic accuracy of the occurrence rate of covert RS of the right lateral SCC for distinguishing USH1 patients from controls was 89,7% (AUC: 0.897), using a threshold of 14%, with a sensitivity of 80% and specificity of 96.7%. The diagnostic accuracy of the latter parameter for distinguishing USH1 from USH2 patients was 85% (AUC: 0.85), using a threshold of 13%, with a sensitivity of 80% and a specificity of 88.9.% (Table 3).

## Discussion

In the current study we investigated VOR dynamics using vHIT to differentiate between Usher subtypes 1 and 2. Our main findings were:

(1) VOR gains of all SCCs were significantly different between USH and controls; (2) USH1 patients showed significantly lower VOR gains than USH2 patients for all SCCs except right anterior SCC; (3) USH2 presented lower VOR gains than controls for the right and left anterior SCCs; (4) VOR gain of the right lateral SCC could discriminate controls form USH1 patients, and USH2 from USH1 patients with an overall diagnostic accuracy of ~ 90%; (5) DHI score negatively correlated with VOR gain for USH1 patients; (6) Refixation covert saccades occurrence rate for horizontal SCCs could discriminate USH1 from USH2 patients and controls with a diagnostic accuracy of ~ 85%.

Vestibular dysfunction using low-frequency stimulation (eg, caloric and rotational responses) has been described in Usher syndrome^14^. Vestibular responses have been said to be normal in USH2 patients, while being classically abnormal in USH1 patients^9,14^. Others have described abnormal caloric responses not only in USH1, but also in USH2 patients^5,14^. Caloric and rotational tests have been recently used to study the horizontal SCCs function in a genetically and ophthalmologically confirmed population of 90 patients with the three types of Usher syndrome. Unfortunately, the latter assessment did not provide information on vertical SCCs function^5^. Moreover, in the latter study, no consistent genotype–phenotype correlations could be found, which might preclude the use of the former type of assessment as a screening method for detecting vestibular dysfunction in infant population, where it would be most useful^5,15^. Therefore, there is the need for the use of less invasive and time-consuming vestibular tests, which could be particularly useful in children with a suspected Usher’s phenotype. Ideally, such instrument could distinguish between USH genotypes, and establish a genotype–phenotype correlation earlier in life^16–20^. Unfortunately, vestibular testing at higher frequencies (eg, by using vHIT), has been scarcely used in USH patients. Magliulo et al. recently described vHIT SCC dysfunction in 10 patients affected by Usher with the anterior semi-circular SCCs being the most affected^11^. However, USH genotype was not provided, thus precluding a deeper understanding on a putative vestibular phenotypical difference between USH1 and USH2 patients. In a later study, the same group studied 5 patients with USH2 genotype (4 USH2A and 1 USH 2C). Here, anterior and horizontal SCCs showed a significant VOR deficit bilaterally in 2 patients, while posterior SCCs related responses were deficitary in 4 patients. The authors concluded that vHIT could provide useful additional information to characterize the vestibular phenotype in patients with USH2^12^. Notably, our vHIT findings in a larger sample of USH patients allowed us to distinguish between patients and controls, and to further discriminate between USH1 and USH2 groups with high diagnostic accuracy. While our findings support the existence of greater vestibular severity in USH1 patients, they also show the presence of a less severe, but nevertheless non-negligible, deficit in USH2 group. Indeed, USH2 patients showed significantly differences from controls for anterior SCCs-related function, and from USH1 patients for all the SCC-related function, except for the right anterior SCC. The lack of a statistically significant difference between the control and USH2 groups in the posterior and lateral SCCs, even when controlling for age bias, may be attributed to our limited sample size. Although we adjusted for age influences, there is a trend in the literature indicating a progressive increase in VOR gain loss with age, and we had a small sample size^21,22^. The existence of distinct embryological development for the anterior SCCs could have additionally played a role for the significant differences between both USH subtypes and controls^23^. Also, even in normal subjects, more variable responses are obtained for anterior and posterior SCCs vHIT responses, when compared with lateral SCCs responses^20^.

Our results on the vestibular phenotype of USH1 and USH2 partially support current evidence originated from disease models of mouse inner ears, based on the function of Usher proteins. Usher proteins are most abundant in the stereocilia and the synaptic regions of hair cells and guide the cohesion and development of hair bundles^24^. Mysoin VIIa (USH1B) (actin-binding molecular motors) and cadherin 23 (USH1D) (cell adhesion molecules) are responsible for the cohesion of the stereocilia, the shaping of the hair bundle and cadherin 23 and protocadherin 15 (cell adhesion molecules) are integral to the structure of the tip links. ADGRV1 (USH2C) (adhesion G coupled receptor) and usherin (USH2A) (cell adhesion molecules) are components of ankle links, filaments connected to the base of stereocilia^24,25^. It is known by studies in mouse models that USH1 proteins are necessary for the correct development of the stereocilia in the type I and type II vestibular hair cells, while loss of USH2 proteins don´t seem to be able to misshape the compact hair bundles of vestibular hair cells at least with the same impact^1^. Nevertheless, our vHIT results raise the suspicion for the existence of relevant impact of these dysfunctional proteins on the mature hair bundle of type I vestibular cells.

Considering clinical correlation between VOR gain and reported symptoms, we could find a lower self-perceived handicap in USH1 than in USH2. Additionally, in USH1 group, we could find a negative correlation between VOR gain and DHI score. As expected, it seems that the presence of greater vestibular deficits, particularly in USH1 patients, tend to have greater chance of being noted, allowing for a stronger correlation between objective and subjective assessments. However, the mismatch between objective and subjective measures found in USH2, has been extensively reported in literature in several other vestibular disorders, highlighting that one single vestibular test might not capture vestibular dysfunction in its entire range, including self-perceived symptoms, and associated emotional and physical distress^26^. In sum, DHI questionnaire seems to be helpful for monitoring vestibular function in USH1 patients.

Importantly, the occurrence rate of covert RS during lateral SCCs testing was also able to differentiate USH1 patients from controls and from USH2, with a high rate of diagnostic accuracy and reasonable sensitivity and specificity. In patients with vestibular loss, RS are described as a compensatory mechanism to minimize the effect of the dynamic VOR deficit and as substitutional mechanism within the oculomotor system^27–30^. As the VOR deficit was greater in USH1 patients, our results are in accordance with the literature. Other authors found that both RS frequency and velocity differentiated patients with and without vestibular loss on vHIT^31,32^. Covert saccades were more frequent and had higher peak velocities in USH1 patients and were almost inexistent in USH2, most probably due to the significantly lower VOR gain in the first group. Overt saccades were also present in USH2, but with lower frequency and velocity peaks than USH1, possibly due to the same reason^33^. Since overt RS are generated to compensate for the visual or position error between initial and final visual axis displacement in a deficient VOR, they are additionally dependent of a central visual trigger mechanism. Considering the visual impairment in USH patients, it's plausible that this deficit might contribute to overt RS outcomes. However, after adjusting for best-corrected visual acuity in our USH1 and USH2 patients, no significant differences were identified in the comparisons between the subgroups, in agreement with the non-significant difference observed for the best-corrected visual acuity levels between the subtypes. This deserves future studies on non-syndromic retinitis pigmentosa patients, since preliminary studies in visually impaired controls, suggest that visual acuity does not significantly influence vHIT outcomes^34^. In our study, the RS were more obviously detected in the lateral SCC plane compared to the vertical canal planes. This has been previously demonstrated in other studies^35,36^.

Our study has several limitations. We included a small number of patients. That was due to the intrinsic rarity of the disease. Still, we used parametric and conservative non-parametric tests with age and best corrected visual acuity as covariates to avoid Type I and type II errors. 6 of our USH1 patients have had one or two cochlear implants, and such procedure could have disturbed the vestibular apparatus^37^. Again, the infrequency of this entity poses a limitation or potential bias to our research, still, none of them complained of vertigo after surgery. Also, there is contrasting anecdotal evidence showing improvement of vestibular function after cochlear implant surgery^38–40^. It's worth noting that USH1 patients typically experience early profound hearing loss, leading to timely investigation and diagnosis^41^. With the implementation of newborn screening programs, these patients are now identified in infancy and ideally undergo cochlear implantation before the age of 2-years-old^42^. Consequently, some of these patients might not have been extensively studied prior to receiving implants. However, as most recent years have seen an increase in studies focusing on vestibular screening in children with profound and severe hearing loss, we anticipate that future research will address this limitation^43^.

The vHIT is an emerging technique to evaluate the six semicircular canals function at high frequencies (5–10 Hz). Numerous studies have validated vHIT for pediatric vestibular assessment^44–46^. In our investigation, which utilized a goggle-based system, adjustments were necessary to accommodate pediatric anatomical and behavioral differences of our two youngest 9-years-old participants. Following the approach of other authors, we raised the right eyelid on the camera side, directed light to the eyes, and placed a sponge in the elastic band to secure the goggles^45,46^. Additionally, a small stool was provided for the children to place their feet and sit upright. Due to these constraints, the LARP plan couldn't be completed in the 9-year-old control participant. However, with the other teenage participants, we encountered no difficulties.

Of note, we found vHIT asymmetries between right and left sides both in in patients and controls. Gain asymmetries for right and left SCCs impulses have been described in the literature in normal individuals and attributed to a greater acceleration in the adducting eye (in our case, the right eye for rightward head impulses) and observer’s right-handedness^20^. Still, we do not believe that the latter findings might have significantly influenced our results.

vHIT seems to be a potentially key screening tool for detecting vestibular dysfunction in USH patients, and further helps to discriminate between USH genotypes. The implications of an early USH diagnosis cannot be overemphasized. This will allow for a timely hearing, vestibular, visual, and social intervention. Importantly, children identified with a deafblind disease before 6 months may acquire normal speech and language delays if treated early. Future multicenter studies performing vHIT in infants are needed to clarify vHIT as a possible clinical biomarker in Usher syndrome.

## Conclusion

vHIT can be an important screening tool used to successfully distinguish between Usher patients and controls in a fast and accurate manner. It may also help to distinguish USH1 from USH2 with important prognostic implications.

## Data Availability

The datasets used and/or analysed during the current study are available from the corresponding author on reasonable request.

## Abbreviations

AUC-ROC: Area under the receiver operating characteristic curve
DHI: Dizziness handicap inventory questionnaire
LARP: Left anterior right posterior
RALP: Right anterior left posterior
SCC (s): Semicircular canal(s)
SNHL: Sensorineural hearing loss
SPSS v.29: Statistical Package for the Social Sciences version 29
USH: Usher patients
USH1: Usher type 1 patients
USH2: Usher type 2 patients
USH3: Usher type 3 patients
USH4: Usher type 4 patients
RS: Refixation saccades
vHIT: Video Head Impulse Test
VOR: Vestibulo-ocular-reflex

## References

[1] Delmaghani S, El-Amraoui A (2022). The genetic and phenotypic landscapes of Usher syndrome: From disease mechanisms to a new classification. Hum. Genet..

[2] Cesca F, Bettella E, Polli R, Leonardi E, Aspromonte MC, Sicilian B, Stanzial F, Benedicenti F, Sensi A, Ciorba A, Bigoni S, Cama E, Scimemi P, Santarelli R, Murgia A (2020). Frequency of Usher gene mutations in non-syndromic hearing loss: Higher variability of the Usher phenotype. J. Hum. Genet..

[3] Castiglione A, Möller C (2022). Usher syndrome. Audiol. Res..

[4] Abad-Morales V, Navarro R, Burés-Jelstrup A, Pomares E (2020). Identification of a novel homozygous ARSG mutation as the second cause of Usher syndrome type 4. Am. J. Ophthalmol. Case Rep..

[5] Wafa TT, Faridi R, King KA, Zalewski C, Yousaf R, Schultz JM, Morell RJ, Muskett J, Turriff A, Tsilou E, Griffith AJ, Friedman TB, Zein WM, Brewer CC (2021). Vestibular phenotype-genotype correlation in a cohort of 90 patients with Usher syndrome. Clin. Genet..

[6] Espinós C, Pérez-Garrigues H, Beneyto M, Vilela C, Rodrigo O, Nájera C (1999). Sordera hereditaria sindrómica: Síndrome de Usher. Aspectos otoneurológicos y genéticos [Syndromic hereditary deafness Usher's syndrome. Oto-neurologic and genetic factors]. An. Otorrinolaringol. Ibero. Am..

[7] Sadeghi M, Cohn ES, Kimberling WJ, Tranebjaerg L, Möller C (2005). Audiological and vestibular features in affected subjects with USH3: A genotype/phenotype correlation. Int. J. Audiol..

[8] Teschner M, Neuburger J, Gockeln R, Lenarz T, Lesinski-Schiedat A (2008). "Minimized rotational vestibular testing" as a screening procedure detecting vestibular areflexy in deaf children: screening cochlear implant candidates for Usher syndrome type I. Eur. Arch. Otorhinolaryngol..

[9] Kumar A, Fishman G, Torok N (1984). Vestibular and auditory function in Usher's syndrome. Ann. Otol. Rhinol. Laryngol..

[10] Halmagyi GM, Chen L, MacDougall HG, Weber KP, McGarvie LA, Curthoys IS (2017). The video head impulse test. Front. Neurol..

[11] Magliulo G, Iannella G, Gagliardi S (2015). Usher's syndrome: Evaluation of the vestibular system with cervical and ocular vestibular evoked myogenic potentials and the video head impulse test. Otol. Neurotol..

[12] Magliulo G, Iannella G, Gagliardi S (2017). Usher's syndrome type II: A comparative study of genetic mutations and vestibular system evaluation. Otolaryngol. Head Neck Surg..

[13] Vaz GF, Luzio CS, Benzinho TA, GabãoVeiga V (2008). Validação e adaptação do Dizziness Handicap Inventory para a língua e população portuguesa de Portugal. ACTA ORL/Técnicas em Otorrinolaringologia.

[14] Mathur P, Yang J (2014). Usher syndrome: Hearing loss, retinal degeneration and associated abnormalities. Biochim. Biophys. Acta.

[15] Friedman TB, Schultz JM, Ahmed ZM, Tsilou ET, Brewer CC (2011). Usher syndrome: Hearing loss with vision loss. Adv. Otorhinolaryngol..

[16] Nolen RM, Hufnagel RB, Friedman TB, Turriff AE, Brewer CC, Zalewski CK, King KA, Wafa TT, Griffith AJ, Brooks BP, Zein WM (2020). Atypical and ultra-rare Usher syndrome: A review. Ophthalmic Genetics.

[17] Wiener-Vacher SR, Wiener SI (2017). Video head impulse tests with a remote camera system: Normative values of semicircular canal vestibulo-ocular reflex gain in infants and children. Front. Neurol..

[18] Bachmann K, Sipos K, Lavender V, Hunter LL (2018). Video head impulse testing in a pediatric population: normative findings. J. Am. Acad. Audiol..

[19] Emekci T, Uğur KŞ, Cengiz DU, Men Kılınç F (2021). Normative values for semicircular canal function with the video head impulse test (vHIT) in healthy adolescents. Acta Otolaryngol..

[20] Matiño-Soler E, Esteller-More E, Martin-Sanchez JC, Martinez-Sanchez JM, Perez-Fernandez N (2015). Normative data on angular vestibulo-ocular responses in the yaw axis measured using the video head impulse test. Otol. Neurotol..

[21] Agrawal Y, Berg R, Wuyts F, Wather L (2019). Presbyvestibulopathy: Diagnostic criteria consensus document of the classification committee of the bárány society. J. Vestib. Res..

[22] Welgampola, M.S., Taylor, R.L., Halmagyi, G.M. Video Head Impulse Testing. In: Lea J, Pothier D (eds.): Vestibular Disorders. Adv Otorhinolaryngol. Basel, Karger, vol. 82, pp. 56–66, 10.1159/000490272 (2019) 10.1159/000490272 30947183

[23] Catala M (2014). Embryologie de l’oreille interne. EMC - Oto-rhino-laryngologie.

[24] deJoya EM, Colbert BM, Tang PC, Lam BL, Yang J, Blanton SH, Dykxhoorn DM, Liu X (2021). Usher syndrome in the inner ear: Etiologies and advances in gene therapy. Int. J. Mol. Sci..

[25] Géléoc GGS, El-Amraoui A (2020). Disease mechanisms and gene therapy for Usher syndrome. Hear. Res..

[26] Yip CW, Strupp M (2018). The dizziness handicap inventory does not correlate with vestibular function tests: A prospective study. J. Neurol..

[27] Riska KM, Bellucci J, Garrison D, Hall C (2020). Relationship between corrective saccades and measures of physical function in unilateral and bilateral vestibular loss. Ear. Hear..

[28] Hermann R, Pelisson D, Dumas O (2017). Are covert saccade functionally relevant in vestibular hypofunction?. Cerebellum.

[29] Schubert MC, Zee DS (2010). Saccade and vestibular ocular motor adaptation. Restor. Neurol. Neurosci..

[30] Blödow A, Pannasch S, Walther LE (2013). Detection of isolated covert saccades with the video head impulse test in peripheral vestibular disorders. Auris. Nasus Larynx..

[31] Rambold HA (2016). Age-related refixating Saccades in the three-dimensional video-head-impulse test: Source and dissociation from unilateral vestibular failure. Otol. Neurotol..

[32] Janky KL, Patterson J, Shepard N, Thomas M, Barin K, Creutz T, Schmid K, Honaker JA (2018). Video head impulse test (vHIT): The role of corrective saccades in identifying patients with vestibular loss. Otol. Neurotol..

[33] Yacovino DA, Martin LA, Perez Akly M, Hain TC (2018). Characteristics of vestibular corrective saccades in patients with slow visual saccades, vestibular disorders and controls: A descriptive analysis. PLoS One.

[34] Judge PD, Rodriguez AI, Barin K, Janky KL (2018). Impact of target distance, target size, and visual acuity on the video head impulse test. Otolaryngol. Head Neck Surg..

[35] Cremer PD, Halmagyi GM, Aw ST, Curthoys IS, McGarvie LA, Todd MJ, Black RA, Hannigan IP (1998). Semicircular canal plane head impulses detect absent function of individual semicircular canals. Brain.

[36] Psillas G, Petrou I, Printza A, Sfakianaki I, Binos P, Anastasiadou S, Constantinidis J (2022). Video head impulse test (vHIT): Value of gain and refixation saccades in unilateral vestibular neuritis. J. Clin. Med..

[37] Koyama H, Kashio A, Fujimoto C, Uranaka T, Matsumoto Y, Kamogashira T, Kinoshita M, Iwasaki S, Yamasoba T (2021). Alteration of vestibular function in pediatric cochlear implant recipients. Front. Neurol..

[38] Guan R, Wang Y, Wu S, Zhang B, Sun J, Guo X, Sun J (2021). Vestibular function in children and adults before and after unilateral or sequential bilateral cochlear implantation. Front. Neurol..

[39] Ibrahim I, da Silva SD, Segal B, Zeitouni A (2017). Effect of cochlear implant surgery on vestibular function: Meta-analysis study. J. Otolaryngol. Head Neck Surg..

[40] Wang R, Luo J, Chao X, Wang H, Fan Z, Xu L (2022). Minimally invasive surgical techniques in vestibular function preservation in patients with cochlear implants. Front. Neurosci..

[41] Nisenbaum E, Thielhelm TP, Nourbakhsh A, Yan D, Blanton SH, Shu Y, Koehler KR, El-Amraoui A, Chen Z, Lam BL, Liu X (2022). Review of genotype-phenotype correlations in usher syndrome. Ear. Hear..

[42] Purcell PL, Deep NL, Waltzman SB, Roland JT, Cushing SL, Papsin BC, Gordon KA (2021). Cochlear Implantation in Infants: Why and How. Trends Hear..

[43] Martens S, Maes L, Dhondt C, Vanaudenaerde S, Sucaet M, De Leenheer E, Van Hoecke H, Van Hecke R, Rombaut L, Dhooge I (2022). Vestibular infant screening-flanders: What is the most appropriate vestibular screening tool in hearing-impaired children?. Ear. Hear..

[44] Wiener-Vacher SR, Wiener SI (2017). Video head impulse tests with a remote camera system: Normative values of semicircular canal vestibulo-ocular reflex gain in infants and children. Front. Neurol..

[45] Bachmann K, Sipos K, Lavender V, Hunter LL (2018). Video head impulse testing in a pediatric population: Normative findings. J. Ame. Acad. Audiol..

[46] McGarvie LA, MacDougall HG, Halmagyi GM, Burgess AM, Weber KP, Curthoys IS (2015). The video head impulse test (vHIT) of semicircular canal function - age-dependent normative values of VOR gain in healthy subjects. Front. Neurol..

